# Indents

**Published:** 1914-05-15

**Authors:** 


### INDENTS.

☞Brief Articles of Technical or Scientific Value will receive notice and comment. Contributions invited.

*Medical Education in the United States.* *The Scientific American*, quoting from an interesting report on this subject by Graham Lusk, published in *Science*, states that in spite of the campaign for higher standards waged by the Council on Medical Education of the American Medical Association and other agencies, there are still four states in which it is not necessary that an applicant for medical license be a graduate of a reputable medical college, while in the year 1912 the authorities of Tennessee presented the spectacle of licensing 175 persons (including 33 who were granted temporary licenses after having failed to pass the required examination) who were not graduates of any medical school whatever. As to the medical schools themselves, however, it is gratifying to learn that quality has improved at the expense of quantity. Lusk gives the number of schools in this country as diminished from 166 to 110 since 1904. (The correct figures should show a diminution of medical schools in this country from 160 to 106 since 1904.)—*Journal A. M. A.*

*Dental Dispensary for Albany Schools.* The city of Albany, New York, has developed an excellent system of "health direction in the public schools," which it it is the aim of the medical director to extend considerably beyond the usual concept of mere medical inspection of school children. Recently a dental dispensary has been added which aims to take care of the teeth of children who are not able to pay for the services of a dentist. A set of card-blanks has been provided for keeping a record of the dental work done for the pupils. One of the cards contains sociological data concerning the family from which the pupil comes. The cards given to the children recording appointments and granting admission to the dispensary contain practical admonitions to the pupil and parents as to the importance of taking care of the teeth, their influence on the physical and mental development and the bearing of proper development on future success in life, all of which should have a wholesome educational influence. The school work in Albany is an example of how a scientific foundation along lines of school hygiene can be made successful through the co-operation of progressive citizens, trained educators and child hygienists. The dental dispensary is not the least valuable department of this feature of the health work in the public schools.—*Journal A. M. A.*, March 7, 1914.

*Local Anesthetic Without Cocain.* The following is recommended as a local anesthetic for mucous surfaces:—

| | |
|---|---|
| Phenol | gr. xxx |
| Menthol | gr. xxx |
| Quininae hydrochloratis | gr. xxii |
| Adrenalin puri | gr. 1-12 |

This forms a syrupy liquid, a few drops of which applied by means of a cotton swab to the parts to be anesthetized immediately causes the mucous membrane to become pale; it contracts and is insensitive. It is not caustic, and serves a good purpose in cases where cocain cannot safely be used. —*A. M. A.*

*Care of Deciduous Teeth.* The use of copper cement in the fissures and of plaque destruction by means of ribbon floss and some abrasive powder between the deciduous molars is practically all we need to start with the care of the little child's teeth, provided we get them early enough.—*Caulk's Quarterly.*

*Arresting Hemorrhage Following Extraction.* In post-operative bleeding a tampon of cotton saturated in a five per cent. solution of iodin in chloroform is introduced into the empty alveolus, inducing prompt arrest of the hemorrhage.

*Applying Cement.* In cementing an inlay, crown or facing, dentists can profit by the precept of the cabinet maker who uses the least possible amount of glue that can be spread to thoroughly cover the surface. Dental cements employed as adhesives should follow the application of the same law of physics—spread it thin.—*Caulk's Quarterly.*

*An Anti-Oxidizer.* Where casting is to be done against an alloyed metal, oxidation can be prevented by painting the surface to be cast against with a fluid consisting of equal parts of saturated solution of borax and saturated solution of boracic acid each in alcohol. Apply with a camel's hair brush and hold the piece in a flame to burn out the alcohol.

*Laying up Trouble.* Those manufacturers who are recommending the use of a hot air blast to hasten the setting of silicate cements are inviting trouble. Did you ever notice a pavement of Portland cement being laid on a hot day? Unless it is sprinkled occasionally it checks and may even explode. Most dental cements are the same in principle. If they are carefully balanced, they must be kept free from both air and outside moisture while setting.—*Caulk's Quarterly.*

*Oral Sepsis the Cause of Heart Trouble.* Dr. J. M. Lewis, in the *Austrian Journal of Dentistry*, relates a case of a patient suffering severely from septic endocarditis who was relieved by the extraction of several upper incisors which had been crowned. The roots were not abscessed, but the gum around them were of a purple color, and the crowns favored the lodgment of food debris. Following the extraction of the roots the heart condition cleared up and the patient enjoyed a long immunity from the attacks.

*Mouth Disinfection with Ultraviolet Rays.* Dr. Freidburger and Shioji, in the *Deutsche Wochenschrift*, of Berlin, report the results of a research which strongly confirms the strong bactericidal action of the Ultraviolet rays. When applied inside the mouth of a rabbit previously infected with various germs, the germs were rapidly killed. They suggest the possibility of eradicating diphtheria bacilli from the throats of common carriers. Also the sterilization of the mouth and teeth.—*Journal A. M. A.*

*Medical Schools and Graduates.* There are at present 106 Medical Colleges in the United States and last year they graduated 3981 doctors. There has been a steady falling off of graduates since 1904, when there were 5747 medical graduates. Since the examination of school by the Carnegie Foundation a few years ago, and the classification by grades of all schools, there has been a marked decrease in the number of schools. About thirty State Examining Boards do not now recognize such schools as have the "C" grade. There are 142,000 medical practitioners in the United States, this gives each practitioner about 700 patients to look after.

*Medical Endowment.* James E. Deering, of Chicago, has just given a million dollars to Wesley Memorial Hospital, the interest of which is to be used to support the teachers in the Northwestern Medical School and other medical benevolences through this hospital and school. This ought to help make this one of our great teaching schools.

*Expensive Sandwich.* Anyone who observes carefully the eating habits of working men in this country and who has followed the enormous increase in the lunch-counter scheme of dietetics among our own population must admit that the sandwich is something more than a trivial incident in the nutrition of those who live in populous districts. Physiologically, it involves the supplementing of bread —the common "staff of life"— with considerable fat (butter) and animal protein (meat). The sandwich represents a new step in the evolution of bread-and-butter combinations. Rubner believes that the growing use of the meat-laden sandwich is attributable, in Germany at least, to the increased employment of tea and coffee, which require some substantial adjuvant, and also to the greater consumption of sugar and alcohol. The latter lead to a lowering of the protein of the diet which is thus equalized by the albuminous sandwich. Added to these factors is the growing tendency, especially among the unmarried classes, to eat outside of the home and to patronize the rapid-service, time-saving, sandwich-dispensing restaurants and eating-houses.

The average composition of the sandwich, if we may identify this for the purpose of argument with its German competitor, shows that it differs from plain bread in the predominant addition of fat with some increase in protein. One can attach but little value to the "average" composition of so heterogeneous a group of products as is represented by bread-and-meat mixtures of all varieties. Broadly speaking, the comparison afforded shows that 100 calories are distributed in the different food materials as follows:

| | Calories | | |
|---|---|---|---|
| | Protein | Fat | Carbohydrate |
| In bread | 11 | 3 | 86 |
| In bread and butter | 5 | 58 | 37 |
| In meat sandwiches | 15 | 53 | 32 |

The great concentration of nutrients in a small volume in the sandwich at once becomes apparent here. The work of mastication is reduced and the entire make-up of the product encourages rapid eating with its possibly unfavorable consequences.

The sandwich as here represented, exemplifies a tendency to increase rather than diminish the proportion of food of animal origin in the dietary of man; but aside from the fact that it is instituting a larger participation in the use of meat and is thus working contrary to what many students of dietetics regard as desirable, this form of food is not as economical as is popularly believed. It is true that a palatable sandwich can be purchased for a few cents. The same proportionate expenditure in the household or in the purchase of a warm meal that deserves the name will procure surprisingly more nutriment, even in the more expensive type of restaurant. It has been calculated, for example, that twenty-five cents will buy:

| | Calories | | Gm. protein |
|---|---|---|---|
| In a public eating-house | 3,990 | containing | 108 |
| In a good restaurant | 1,990 | containing | 78 |
| In the form of sandwiches | 1,140 | containing | 30 |

The sandwich is frequently looked upon as the "poor mans' lunch" and current practice is tending to increase its use. If it is really desirable to increase the purchasing power of a small daily income so as to augment the part devoted to nutriment, the reform cannot be instituted by pointing to the supposedly inexpensive lunchcounter. The boarding-house and the home wisely administered on the dietetic side still remain the most economical as well as most rational centers for food reforms.—*Journal A. M. A.*, March 7, 1914.

*Arsenic in Temporary Teeth.*

I think it is a pretty safe rule to figure somewhat along this line; that the resorption of the root of a temporary tooth takes about three years. In other words, if the deciduous centrals are lost at seven, the resorption of the root starts at about four, and if you have to devitalize, it will not be well to use arsenic in that tooth in a child five or six years old, because at that time there is considerable resorption. If you have to devitalize a central incisor before the child is four years of age you are fairly safe in using arsenic. Take a second temporary molar usually lost, between 10 and 11, three years previous to that root resorption begins, a child about six years of age, you are safe in using arsenic, but in a child of eight and a half or nine years of age I would not like to use it.—*G. W. Dittmar, in Dental Review.*

*Experimental Evidence of Variations in Alimentary Secretions and their Pathological Results.*

Under this head Prof. H. P. Pickerill, of Otago, New Zeland, publishes in the January, 1914, number of the *Interstate Medical Journal* of St. Louis, Mo., a very instructive article dealing with his favorite theme "Salivary Secretion." In his "Cartwright Essay," published several years ago he pointed out the effect of various food materials on the salivary flow, and its relation to the incidence or inhibition of dental caries. He showed, contrary to all preconceived ideas, that acid fruits were inhibitory to caries, arguing that the salivary stimulation induced by them tended to bring about a condition of immunity.

He now goes further and, after a study of the action of various foods on the alimentary canal, he says; "I desire to show that *what is best for the mouth is best for the remainder of the alimentary tract;* that, in fact, the several digestive cavities are governed by identical or similar laws, and further, from the point of view of initiating and maintaining normal alimentary secretions and peristalsis, the mouth is *the most important part of the whole canal.*" He further says: "It is important to recognize that salivary secretion is normally a reflex one, and that the normal stimulant to the flow is the food we eat, and, as I propose to show, differences in food produce enormous differences in the character of the juice secreted."

He then goes on to show the effects of various food materials on the functions of the alimentary tract, and recites experiments which prove the desirability of employing such foods as shall stimulate salivary secretion. The article should be read by dentists as well as by medical men.—*C. N. Johnson, in Review.*

*The Ductless Glands and the Teeth.* Arnold Josefson, of Stockholm, in *Deutsche Arch. fur Klinical Medicine,* asserts that dentition and the early development of the hair are under the control of some of the ductless glands. He cites cases of record where disturbance in normal dentition were associated with known defects in the function of glands of internal secretion, and also that such anomolies can be corrected by suitable organotherapy. He cites cases of delayed dentition which have been helped by the administration of thyroid extract. We are wondering if there may not also be found here a reason for delayed or interrupted development of the jaws, with consequent irregularities in second dentition. Kirk thinks there may be a relation to the salivary content accounting for the periods of immunity and decay of the teeth. Almost all obscure diseases have from time to time been referred to the pituitary, thyroid and other so called ductless glands, and it may be that the newer forms of medication may open up to us the real functions of these mysterious organs, and give us new remedies for combatting disease.

*Caustic Applicators.* The Antiseptic Supply Co. of New York, has put on the market useful appliances in the form of wooden sticks tipped like matches with silver nitrate, copper sulphate, alum etc., to be used but once to apply the cautery or styptic definitely without danger of infection or other injury. They should be very useful in the dental office.

*Cleanliness in Serving Food.* The New York Civic Federation has started a campaign of investigation of the manner in which food is prepared and served in public eating houses. Civic inspection of foods, like milk, meat, etc., is of little value if it is contaminated by our cooks and restaurant waiters. We once had an opportunity of witnessing the manner of serving food on a dining car, and we have since then made our choice when ordering meals of such articles as would be least likely to be contaminated by the necessary handling with unclean hands during the process of serving. Baked potatoes, boiled eggs, tea, etc., can not readily be unclean. We also have often wondered what avail it that our food come to the table pure and undefiled only to be saturated with oral filth before the digestive juices have a chance to convert it into healthy pabulum. Perhaps if we wait long enough, we shall get to the oral cavity as a real source of danger, and perhaps some benevolent organization will cause the people to at least take as much care to have the mouth clean before eating as well as the hands, dishes and food.

*Tobacco Smoking and Mental Efficiency.* Dr. Bush, in the *New York Medical Journal* of March 14, shows that in a test of fifteen men smoking reduced mental efficiency ten per cent. In the field of imagery there was a loss of 22 per cent. Ciagrettes were most offensive. Nicotine was not found in any of the smoke, but pyridine was present in all of them and seems to be the toxic factor.

*Too many Doctors in Austria.* The Medical Faculty of Vienna University has issued a warning to men contemplating a medical course. The claim is that the profession is overcrowded now and that the constantly increasing number of medical students is not warrented. There is no more room in the medical schools, and there is no present or prospective demand for more doctors, and the present financial outlook for the doctor is anything but inviting. The new national sickness insurance law will seriously effect the incomes of medical practitioners. Why do not some of these surplus physicians turn to dentistry? There is certainly room for more dentists in Austria. Preventive medicine of the future will depend largely on how the people's teeth are conserved. Austria as well as the United States of America needs a lot more dentists, and they should be teachers rather than technicers as is too much the case at present.

*Annual Dental Registration Fees.* Several states have passed laws requiring the payment annually to the State Examining Board of a license fee. We note that in some places the Boards are experiencing considerable trouble in collecting the fee, largely because of the neglect of the dentists. Although the penalties are severe, it costs a lot of time and money to make the collections or penalties, and the Board members do not have the time or inclination to do this kind of work. It will probably resolve itself into the payment of a larger fee with longer periods, or the employment of a collecting agency of some sort to take care of the work, and then the costs will absorb all the revenue.

*Pulp Extirpation.* Cut a piece of elastic rubber tubing about three fourths of an inch in length of a size that will fit tightly the tooth to be operated. Remove the needle from the hypodermic syringe and fit the piece of rubber tubing to the syringe and tie it tightly to place. Then fit the other end of the rubber tubing over the tooth to be operated and by making suction with the syringe plunger draw the blood out of the pulp. If there is no exposure of the pulp one should be made before the rubber and syringe is applied. The aspiration of the blood will not be painful, and the pulp can be removed at once without pain.—Dr. Williams, *in Dental Review.*

*Open the Window.* This is the season when it is most necessary to preach the cult of the open window. Windows open themselves naturally in the summer. We all sigh then for the cool breeze which the high-flung sash invites. But it is so easy in the winter to leave a window shut and keep out the cold. Yet this is precisely the season when the house air is most dangerous.

A window down at the top is seldom a draught-creator. This is a simple fact which many do not know, but a knowledge of which might lead hosts of timid people into lowering the window. Yet nothing draws off the stale air from a room like a window dropped an inch or two from the top. It is far more effective than a window raised at the bottom. And it is the raised window which floods you with cold air and drives you to quickly shut it up.

Air seems to have little difficulty in getting into a house if you will make room for it. There are chinks at the doors which will let it in once a vacuum is created inside by letting the super-heated and foul air out at the top of a lowered window. The upper openings in a house appear to be hermetically sealed when they are closed; while the lower openings fit loosely. This is why a lowered window will draw plenty of fresh air into a room from elsewhere.

One of the developments of modern dress seems to be to dress for a hot house winter atmosphere; and then to put on very heavy wraps to go out. The effect of this is, of course, that our houses are overheated to keep us warm when we sit inactive, with our outside garments laid off. This is about as bad a thing as we could do for health. We breathe baked air all winter—we keep the windows shut to maintain a high degree of heat—we fear an outside current of air when we are not clad for out-of-doors. It would be far better to follow the English custom of dressing more heavily for the house and weighing ourselves down with few outer garments when we go out.

At all events, keep the window open. It prevents tuberculosis—it alleviates catarrh—it enriches the blood—it enlivens the spirits—it tones up the health—and it puts one in better form to resist the attacks of disease. It is the cheapest health receipt open to universal use. The window should be open more hours than the drug store.—*Oral Health.*

*Inhibitive Anesthesia.* Dr. W. J. Hogan, in the *Summary* for April describes a method of producing anesthesia of local areas by pressure on the nerve trunks between the source of nerve influence and the locality to be affected. The method is a suggestion by Dr. Wm. H. Fitzgerald, a rhinologist of Hartford, Conn. The pressure can be applied by means of the thumb and fingers, as at the mental orifice in the maxilla, or by pressure on the lingual opposite the lower third molar, or by a roll of cotton on a probe in the internal nose. In fact at any point where the pressure can be applied with a sufficient degree of force with comfort to the patient, for, from one to two minutes, the peripheral distribution of that nerve can be obtunded.

It is an interesting discovery, and we have no doubt that by a careful study of the anatomy of the oral cavity, it may be made a useful practice for many operations. The reporter describes a case where a considerable number of upper teeth were painlessly extracted by the use of this method. The obtundent effect will sometimes last for an hour or so, but it usually passes in a few minutes. It is claimed that by firm pressure on the apical region in some cases, will so effectually obtund the sensitiveness that a carious cavity may be excavated with entire comfort to the patient. We do not believe this will answer all the demands of a local or general anesthetic but it is a good thing to know about and many times it will help to control a troublesome case. We do not suppose it would answer for a prolonged operation like the extraction of an impacted third molar, or the opening and curettment of an antrum. And we doubt if it could be successfully employed where there was a considor-hemorrhage, such as takes place in operating for pyorrhoea.

